# Assessment of the quality and reliability of YouTube videos related to teleradiology in musculoskeletal and rheumatic diseases: a cross-sectional study

**DOI:** 10.1007/s00296-025-05831-5

**Published:** 2025-03-14

**Authors:** Yerlan Yemeshev, Dana Bekaryssova, Burhan Fatih Kocyigit

**Affiliations:** 1 Radiology Department, South Kazakhstan Medical Academy, Shymkent, Kazakhstan; 2 Department of Biology and Biochemistry, South Kazakhstan Medical Academy, Shymkent, Kazakhstan; 3 Department of Physical Medicine and Rehabilitation, University of Health Sciences, Adana City Research and Training Hospital, Adana, Türkiye

**Keywords:** Internet, Social media, Teleradiology, Musculoskeletal system, Rheumatic diseases, Arthritis

## Abstract

Teleradiology, the remote evaluation of medical images using digital communication technologies, has altered healthcare delivery, particularly in musculoskeletal and rheumatic disorders. This article aims to assess YouTube videos regarding teleradiology in these disorders. The video inspection was conducted on January 15, 2025, using the search terms “teleradiology musculoskeletal system,” “teleradiology rheumatic diseases,” “teleradiology rheumatology,” and “teleradiology arthritis”.A total of 200 videos were evaluated. Total views, likes and comments, video duration, time since upload, and daily engagement metrics were recorded. The videos were categorized based on their origin. The evaluation tools utilized to analyze each video were the Global Quality Scale (GQS), Modified DISCERN Questionnaire, JAMA Benchmark Criteria, and Patient Education Materials Assessment Tool for Audio/Visual Materials (PEMAT-A/V). Of the 44 videos, 20.4% (*n* = 9) were of low quality, 27.3% (*n* = 12) were of intermediate quality, and 52.3% (*n* = 23) were of high quality. Physicians (81.8%) and nonprofit organizations (66.7%) were the predominant producers of high-quality videos. In contrast, health-related websites (29.4%) and nonacademic healthcare institutions (28.6%) constituted the primary sources of poor content. The quality groups showed significant differences in daily views and likes (*p* < 0.05), with high-quality videos receiving the highest engagement. Significant correlations were detected between video duration and content assessment tools (*p* < 0.01). The current results emphasize the need to select high-quality videos from reliable sources such as physicians and nonprofit organizations. Considering YouTube’s extensive use as a source of medical information, it is essential to advocate for expert-driven, high-quality content to augment its educational efficacy.

**Supplementary Information:**

The online version contains supplementary material available at 10.1007/s00296-025-05831-5.

## Introduction

Radiology is essential for diagnosing and treating musculoskeletal (MSK) disorders and rheumatic diseases. These disorders frequently have intricate clinical symptoms, necessitating imaging techniques including X-ray, ultrasound, computed tomography (CT), and magnetic resonance imaging (MRI) for precise evaluation [1, 2]. Timely and accurate imaging assessment is crucial for prompt intervention, disease monitoring, and treatment modifications [3]. Due to the widespread occurrence of MSK and rheumatic conditions, radiologic competence is essential for enhancing patient outcomes.

Teleradiology, the remote assessment of imaging data via digital communication technology, has transformed healthcare provision, especially in MSK and rheumatic disorders. This method allows radiologists to offer expert assessments across various locations, thus facilitating prompt diagnosis and consulting [4]. It is especially beneficial in rural or underserved regions where skilled radiology facilities may be scarce. Teleradiology promotes the efficiency of MSK and rheumatic disease management by permitting rapid image sharing and multidisciplinary collaboration, boosting patient access to expert care [5, 6].

Digital platforms are becoming integral to investigation, instruction, and clinical practice, providing novel opportunities for knowledge distribution, expert cooperation, and patient engagement [7]. The Internet has become an important source of health-related information, with platforms such as YouTube providing easily available content for healthcare professionals and patients [8, 9].

YouTube offers a comprehensive collection of videos on medical subjects, encompassing radiology and teleradiology, featuring visual demonstrations and expert analyses [10]. The use of YouTube as a platform for distributing health information is increasingly supported by medical professionals and experts, as it provides a broadly accessible platform for education and public awareness [11]. The quality and reliability of health-related content on YouTube fluctuate significantly, requiring a thorough evaluation of its content. Comprehending the advantages and constraints of YouTube as a collection of medical information is crucial for its efficient use in education and clinical settings [12, 13].

The growing use of YouTube as an educational tool highlights the necessity of assessing its content, particularly in specialized areas like teleradiology in MSK and rheumatic disorders. A critical gap persists in comprehending the quality and reliability of teleradiology videos in these settings. The intricacy of MSK and rheumatic imaging can result in misinformation or poor content that could mislead consumers, affecting therapeutic decision-making. The current research assesses YouTube videos regarding teleradiology in MSK disorders and rheumatic diseases. The first step is to investigate the basic characteristics of the videos. A key focus is on determining the most reliable resources that should be favored to obtain high-quality content. Furthermore, the study intends to provide helpful understanding by comparing video metrics across quality categories. Finally, it aims to evaluate YouTube’s efficiency as a tool for sharing information on teleradiology in MSK and rheumatic disorders.

## Methods

The video inspection was performed on January 15, 2025, utilizing the search phrases “teleradiology musculoskeletal system,” “teleradiology rheumatic diseases,” “teleradiology rheumatology,” and “teleradiology arthritis.” The query terms were chosen according to Medical Subject Headings (MeSH) to guarantee a systematic and thorough search.

All cookies and browser history were purged before the search to minimize the impact of individualized outcomes. The search was conducted in incognito mode via the Google Chrome browser to ensure anonymity. Videos were organized according to YouTube’s standard “relevance-based ranking” configuration, emulating the conventional user experience [14, 15]. A standard web consumer interacts with only a limited subsection of the query outcomes, a pattern corroborated by prior research. Consequently, the first 50 videos from each search query were chosen for assessment to represent typical user behavior [16, 17].

The subsequent exclusion criteria were implemented to guarantee the selection of relevant content:

- Non-English content.
- Redundant entries.
- Extraneous material of inappropriate length.
- Technical difficulties– Videos exhibiting substandard audio quality, distorted imagery, or other problems that impair understanding.

Videos below 1 min were omitted, as they frequently lack the depth and complexity required for delivering substantial educational content. Shorter videos frequently offer only superficial summaries, advertising collateral, or partial elucidations, rendering them insufficient for thorough information dissemination. On the flip side, videos above 60 min were omitted to conform to standard YouTube user engagement trends and keep the content remains accessible and pragmatic.

The assessment procedure followed a systematic, multi-phase methodology to guarantee precision and dependability. Initially, two investigators independently evaluated and rated each material. Their assessments were subsequently analyzed to detect any discrepancies. In instances of disagreement, a third researcher performed a last review and rendered a conclusive judgment. Cohen’s kappa coefficient was computed to assess the consistency of the initial ratings, offering a statistical measure of agreement [18].

### Video characteristics

The following main variables were directly obtained from YouTube:

- Engagement metrics - Total count of views, likes, and comments.
- Video duration - Quantified in seconds.
- Duration since upload - The number of days from the upload date to the evaluation date.
- Daily engagement metrics - Average daily views, likes, and comments.

For content structure analysis, videos were classified into four distinctive formats:

- Narration-centric - Involving a sole speaker delivering elucidations.
- Patient experience-oriented - Highlighting testimonials or actual case studies.
- Animated content - Employing digital animations to elucidate topics.
- Slide-based presentations - Utilising text and graphics in a slideshow style.

The radiologic methods focused on in the videos were recorded as follows:

- X-ray (Radiography).
- MRI.
- CT.
- Ultrasound.
- Scintigraphy.
- Others.

Additionally, conditions and disorders addressed by radiologic methods in the videos were noted.

### Video sources

Videos were classified according to their source of origin. The subsequent classifications were employed:

- Physician.
- Nonphysician health care professional.
- Academic medical centers.
- Nonacademic healthcare facilities.
- News.
- Nonprofit associations.
- Independent user.
- Health-related websites.

### Content assessment

The Global Quality Scale (GQS), a validated instrument for appraising the educational merit of online materials, was employed to measure video quality. The GQS evaluates content using a five-point scale, with 1 being the lowest quality and 5 the highest [19, 20].

*Low-quality (Scores 1–2):* Videos in this category exhibit substantial inaccuracies, inconsistencies, or omissions, rendering them ineffective for instructional purposes.

*Intermediate-quality (Score 3):* These videos provide some instructional value but potentially lack depth, clarity, or thoroughness.

*High-quality (Scores 4–5):* Videos in this category are meticulously organized, accurate, and instructive, rendering them helpful instructional assets.

Researchers utilized the modified DISCERN instrument to evaluate the reliability of YouTube videos. This instrument assesses various critical elements, including clarity, comprehensibility, bias, objectivity, and the availability of references or other resources. Each criterion is evaluated using a binary system, whereby a positive response is awarded one point, and a negative response receives zero points. The greatest attainable score is 5, signifying excellent reliability [21].

The JAMA Benchmark Criteria offer a systematic framework for evaluating the precision and quality of digital medical knowledge. This approach assesses crucial factors that enhance the legitimacy of digital content, including ownership, authenticity, transparency, and currency. By analyzing these key components, the criteria assist in assessing the reliability of online medical information [22].

The Patient Education Materials Assessment Tool for Audio/Visual Materials (PEMAT-A/V) offers a systematic approach to assessing the efficacy of visual and auditory medical educational resources. This instrument evaluates two fundamental dimensions: understandability and actionability. Understandability assesses the ease with which users can process and grasp information, considering elements such as clarity, organization, phrases, and imagery. Actionability assesses whether the text offers explicit, actionable strategies individuals can implement to handle their disease. Scores are shown as percentages, reflecting the overall standards of the educational material for accessibility and actionability [23].

### Statistical analysis

Statistical analysis was performed using SPSS software (version 29.0, SPSS Inc., Chicago, IL, USA). Before analysis, the Shapiro-Wilk test was conducted to evaluate the normality of data distribution. Descriptive data were presented with medians, frequencies (n), and percentages (%).

The Kruskal-Wallis test was employed to compare across three predetermined categories. The Spearman’s rho test was used to examine correlations among variables. The Kappa coefficient was computed to assess inter-rater agreement. A p-value of less than 0.05 was deemed statistically significant.

## Results

**Fig. 1:**
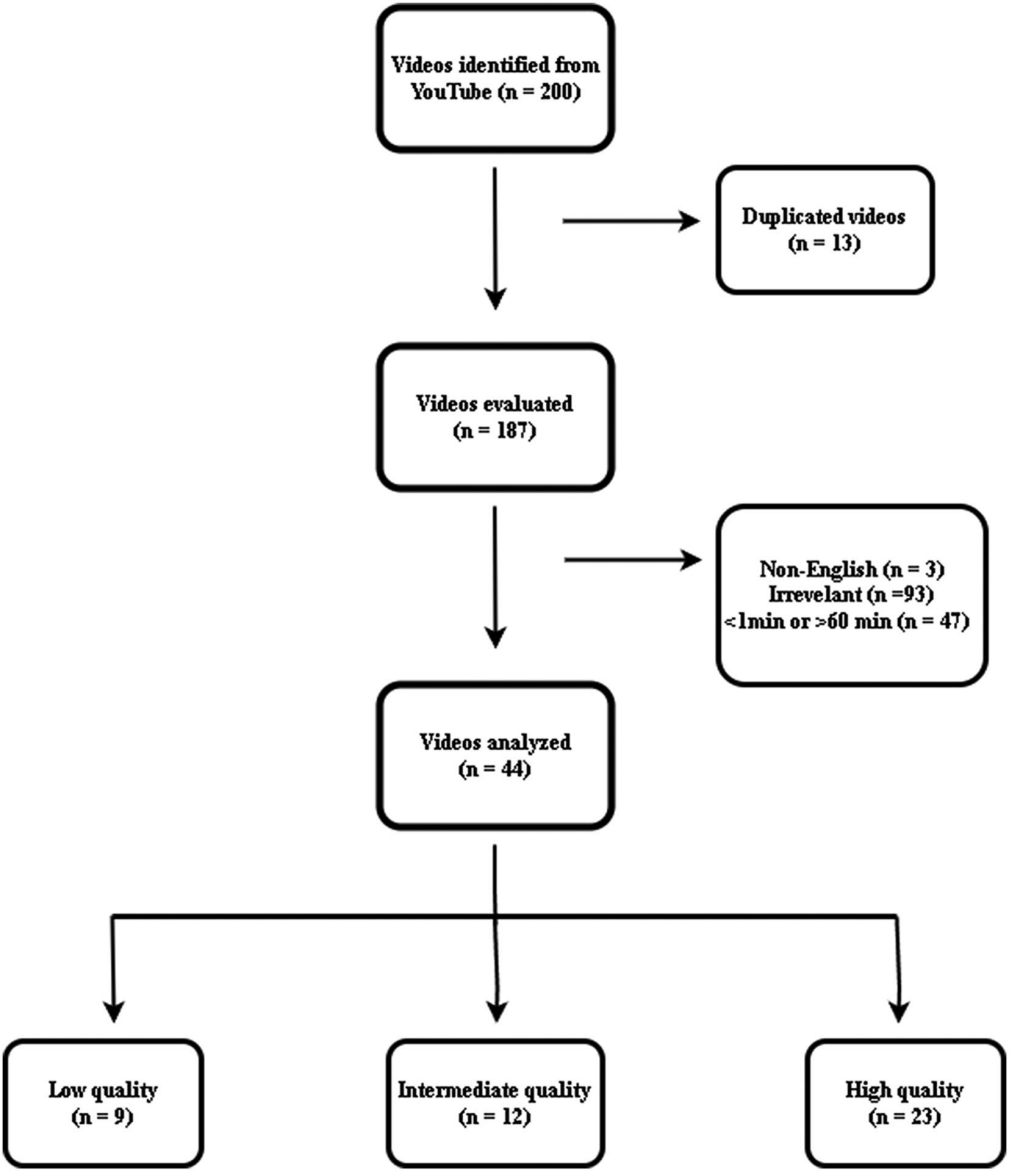
Visualization of the inclusion and exclusion process of videos

A total of 200 videos were initially chosen, comprising 50 videos for each search query according to relevancy. After implementing the predetermined exclusion criteria, 156 videos were discarded, yielding a final collection of 44 videos for analysis. Figure 1 illustrates a detailed outline of the selection procedure. The median duration of the examined videos was 498.50 (61–3301) seconds. The median engagement metrics were as follows: views 2253 (46–58961), likes 15.5 (0–1300), and comments 0 (0–65). When classified by format, the predominant type of videos (68.2%, *n* = 30) utilized slide-based presentations, whilst 25% (*n* = 11) included narrative devoid of visual assistance. Videos including animations constituted 4.5% (*n* = 2), while those highlighting patient experiences represented the most minor proportion at 2.3% (*n* = 1). A detailed analysis of the different video attributes is shown in Table 1.

**Table 1:** Primary features of the videos

Video features	
Duration (seconds)^*^	498.50 (61–3301)
Number of views^*^	2253 (46–58961)
Number of likes^*^	15.50 (0–1300)
Number of comments^*^	0 (0–65)
Days since upload^*^	1900 (261–5484)
Views per day^*^	1.25 (0.02–84)
Likes per day^*^	0.01 (0–0.64)
Comments per day^*^	0 (0–1.69)
**Presentation method (n; %)**	
Video containing only narrator(s)	11 (25)
Video containing patient experiences	1 (2.3)
Animation	2 (4.5)
Narrating with a slide presentation	30 (68.2)

^*^ Data are expressed as median (minimum - maximum)

The videos addressed multiple radiologic imaging modalities, with X-ray (radiography) being the most commonly referred (63.6%, *n* = 28). The subsequent modalities were MRI (52.3%, *n* = 23), CT scans (31.8%, *n* = 14), ultrasound (25%, *n* = 11), and scintigraphy (7.8%, *n* = 3) (Table 2).

**Table 2:** Radiologic imaging modalities mentioned in the videos

Radiologic imaging modalities^*^	*n* (%)
X-ray (Radiography)	28 (63.6)
Magnetic Resonance Imaging	23 (52.3)
Computed Tomography	14 (31.8)
Ultrasound	11 (25)
Scintigraphy	3 (7.8)

^*^ Multiple methods can be recorded in a single video

n: number; %: percentage

The videos addressed various MSK and rheumatic disorders evaluated with radiologic approaches. The predominant category discussed was general MSK issues (65.9%, *n* = 29), succeeded by general arthritis (15.9%, *n* = 7). Specific conditions were osteoarthritis (11.4%, *n* = 5), rheumatoid arthritis (9.1%, *n* = 4), and spondyloarthritis (4.5%, *n* = 2) (Table 3).

**Table 3:** Conditions and disorders addressed by radiologic methods in the videos

Condition-Disorder^*^	*n* (%)
Musculoskeletal-general	29 (65.9)
Arthritis-general	7 (15.9)
Rheumatoid arthritis	4 (9.1)
Osteoarthritis	5 (11.4)
Spondyloarthritis	2 (4.5)

* Multiple conditions-disorders can be recorded in a single video

n: number; %: percentage

The GQS scorings classified the videos into three distinct quality levels: low, intermediate, and high. Of the evaluated content, 20.4% (*n* = 9) was regarded as low quality, whilst 27.3% (*n* = 12) was deemed intermediate level. A majority, 52.3% (*n* = 23), satisfied the criteria for high-quality educational content. An examination of video sources indicated that physicians (81.8%) and nonprofit organizations (66.7%) were the predominant producers of high-quality videos. In contrast, health-related websites (29.4%) and nonacademic healthcare institutions (28.6%) constituted the primary sources of poor content. Table 4 offers a thorough overview of video quality from various sources.

**Table 4:** Categorization of the videos according to sources, N (%)

Source	Low quality	Intermediate quality	High quality	Total
Physician Academic medical centers Nonacademic healthcare facilities Nonprofit associations Health-related websites	1 (9.1) 1 (16.7) 2 (28.6) 0 (0) 5 (29.4)	1 (9.1) 2 (33.3) 3 (42.8) 1 (33.3) 5 (29.4)	9 (81.8) 3 (50) 2 (28.6) 2 (66.7) 7 (41.8)	11 6 7 3 17

n: number, %: percentage

Statistical analysis indicated significant differences among the quality groups regarding daily views and likes (*p* < 0.05), with high-quality videos garnering the highest engagement. No statistically significant difference was noted in the daily comments among the groups (*p* > 0.05). A comprehensive comparison of these parameters is provided in Table 5.

**Table 5:** Comparison of the video parameters between the low-quality, intermediate, and high-quality groups

	High quality	Intermediate quality	Low quality	*p*
Views per day	2.63 (0.12–84)	0.65 (0.13–4.01)	0.62 (0.02–1.42)	0.003
Likes per day	0.03 (0–0.64)	0 (0–0.05)	0 (0–0.03)	0.007
Comments per day	0 (0–1.69)	0 (0–0.01)	0(0–0)	0.074

Statistical analysis revealed significant correlations between video duration and all content assessment tools (*p* < 0.01). Nonetheless, no significant correlation was identified between the duration since upload and any of the video content assessment measures (*p* > 0.05) (Table 6).

**Table 6:** Correlation analysis between content scores and video parameters

	GQS	Modified DISCERN Questionnaire	JAMA Benchmark Criteria	PEMAT-A/V Understandability	PEMAT-A/V Actionability
Video duration	**0.635** ^**a**^	**0.667** ^**a**^	**0.616** ^**a**^	**0.645** ^**a**^	**0.547** ^**a**^
Days since upload	-0.046	-0.054	-0.017	-0.058	-0.100

GQS: Global Quality Scale; JAMA: Journal of the American Medical Association; PEMAT-A/V: Patient Education Materials Assessment Tool for Audio/Visual Materials

^a^ indicates *p* < 0.01

Owing to the restricted number of videos, those depicting patient experiences (*n* = 1) and animations (*n* = 2) were omitted from the analysis. The video quality analysis concentrated exclusively on videos with narrators and those employing slide presentations. The analysis indicated that slide presentation-based videos were significantly more prone to provide high-quality content than narrator-only videos (*p* < 0.01) (Table 7).

**Table 7:** Comparison of quality groups according to video presentation method

	Only narrator (s)	Slide presentation	*p*-value
Hıgh quality (n,%)	0 (0)	22 (73.4)	
Intermediate quality (n,%)	5 (45.5)	7 (23.3)	< 0.001
Low quality (n,%)	6 (54.5)	1 (3.3)	
Total (n,%)	11 (100)	30 (100)	

Kappa value is 0.84.

## Discussion

This study targeted to assess YouTube videos regarding teleradiology in MSK and rheumatic disorders, concentrating on video quality, content, and reliability. The analysis disclosed key results: 52.3% of the videos were classified as high quality, 27.3% as intermediate quality, and 20.4% as low quality. Physicians and nonprofit organizations predominantly generated high-quality videos, whereas lower-quality content primarily originated from health-related websites and nonacademic healthcare institutions. The study indicated that slide-based presentation videos tended to be of a higher standard compared to narration-only videos. Significant correlations were identified between video duration and assessment indicators, suggesting that longer videos typically have more thorough and well-structured. Ultimately, videos of higher quality attracted greater involvement in daily views and likes.

The results of the radiologic imaging methods discussed in the videos offer beneficial insights into the emphasis of YouTube content concerning MSK and rheumatic conditions. X-ray (radiography) was the most frequently mentioned imaging modality, comprising 63.6% of the videos (*n* = 28). This corresponds with the prevalent utilization and accessibility of X-rays in clinical settings for diagnosing MSK-rheumatic disorders, as it is frequently the initial imaging modality employed due to its cost-efficiency and availability. X-rays are beneficial in identifying structural abnormalities, fractures, and joint disorders, which accounts for the frequency in educational materials targeted at a wide audience [24, 25]. MRI closely followed X-ray with 52.3% (*n* = 23), highlighting the importance of this advanced imaging modality for assessing soft tissues, joints, and cartilage in MSK and rheumatic disorders. The comprehensive imaging capabilities of MRI render it a helpful instrument for identifying disorders involving arthritis, soft tissue injuries, and other inflammatory conditions, as mirrored by its frequent reference in instructional videos targeted at medical professionals and patients [26, 27]. Furthermore, the various radiologic imaging techniques featured in the videos demonstrate the depth of diagnostic procedures used in MSK and rheumatic ailments care.

General-MSK scenarios constituted the most common group, featured in 65.9% (*n* = 29) of the videos. This discovery aligns with the extensive occurrence of MSK conditions, which include various disorders impacting bones, muscles, joints, and connective tissues [28]. The prevalence of general MSK l issues indicates that numerous videos intend to furnish fundamental knowledge relevant to a broad audience, encompassing healthcare experts, medical students, and patients seeking general information. The category of general-arthritis was the second most commonly addressed, including 15.9% (*n* = 7). Arthritis constitutes an immense global health issue, profoundly affecting the quality of life and mobility [29]. As indicated by the frequency of arthritis-related videos, YouTube is a resource for individuals seeking to comprehend the radiologic dimensions of joint diseases, including disease progression and monitoring. Osteoarthritis, rheumatoid arthritis, and spondyloarthritis followed these approaches. The data indicate that YouTube information about teleradiology methods for MSK and rheumatic disorders predominantly addresses generic issues, whereas specific diseases are less emphasized. It indicates the necessity to enhance teleradiology content regarding particular diseases.

The evaluation of the videos indicated a diverse quality spectrum, with 52.3% categorized as high quality, 27.3% as intermediate, and 20.4% as low quality. These findings correspond with prior research indicating that the quality of medical content on YouTube can vary considerably [30, 31, 32].The predominance of high-quality videos produced by physicians and nonprofit organizations underscores the significance of expert-oriented content. These sources are more likely to comply with medical standards and deliver precise, evidence-based information—essential in MSK and rheumatic disease-related areas. Conversely, the low-quality content primarily stemmed from health-related websites and nonacademic healthcare institutions. The lower standards of these videos may be ascribed to several factors, including limited subject matter competence, potential commercial interests, or the lack of organized instructional content.

Higher-quality videos earned more views and likes, indicating that customers choose educational and trustworthy material. The lack of a significant difference in comments may indicate that consumers are demonstrating a passive pattern of behavior toward the content. It is critical to recognize that engagement metrics, including views and likes, are not the sole indicator of quality; they may also be influenced by factors such as the producer’s reputation, YouTube’s recommendation algorithms, and the quality of the video production. Although our results indicate a positive correlation between engagement and video quality, they do not establish causation.

The predominant video formats utilized were slide-based presentations (68.2%), followed by narration-centric videos (25%), with fewer animations (4.5%) and patient experiences (2.3%). Videos incorporating slide presentations demonstrated a significantly higher likelihood of high quality than those featuring only narration. This indicates that incorporating visuals with explanations can enhance the clarity and educational effectiveness of the content, facilitating the communication of complex medical information. The positive correlation between video duration and quality assessment tools supports the notion that longer videos typically provide more comprehensive and organized content [33]. Nevertheless, the presumption that longer videos are inherently higher quality is inaccurate. Additionally, short-concise videos can effectively convey high-quality information and emphasize the necessity of balancing the content’s quality and the video’s duration.

Notwithstanding the extensive methodology, several limitations should be acknowledged while interpreting the results. The omission of videos in languages ​​other than English may limit the generalizability of the findings. Considering YouTube’s global reach, future research should analyze non-English videos to offer a more thorough depiction of the available content. Covering the full spectrum of content available on YouTube is impossible with the current methodology. This study offers a snapshot overview of YouTube videos accessible at a particular moment. Due to the platform’s dynamic nature, characterized by the daily upload of new videos and the continual fluctuation of engagement metrics such as views, likes, and comments, the results may differ if the study were repeated at a subsequent date.

Improving the quality of YouTube videos produced by image specialists is essential, ensuring they adhere to enhanced educational standards and deliver correct, trustworthy information for consumers. Moreover, expert opinion surveys in this domain are important for identifying deficiencies and enhancing instructional methodologies [34]. Comprehending the viewpoints of imaging professionals can facilitate the creation of more effective instructional resources, ensuring that the content is up-to-date and consistent with best practices.

## Conclusion

This study offers a valuable assessment of YouTube videos’ quality and instructional merit regarding teleradiology in MSK and rheumatic diseases. The findings emphasize the necessity of choosing high-quality videos from reliable sources, such as physicians and nonprofit organizations. The research indicates that video formats, particularly slide presentations, generally provide higher content quality. While YouTube can serve as an effective instructional resource, users should use prudence and critically evaluate the reliability and accuracy of the videos. The substantial disparity in video quality underscores the necessity for enhanced content regulation, refined editing procedures, and the promotion of reliable sources.

## Electronic supplementary material

Below is the link to the electronic supplementary material.

Supplementary Material 1

Supplementary Material 2

Supplementary Material 3

Supplementary Material 4

Supplementary Material 5

Supplementary Material 6

## Data Availability

Raw data can be provided upon request.

## Declarations

### Disclaimer

Authors state that no part of this manuscript, including the text and graphics, are copied or published elsewhere in whole or in part.

## References

[1] Hamel C, Avard B, Gorelik N et al (2024) Canadian association of radiologists musculoskeletal system diagnostic imaging referral guideline. Can Assoc Radiol J 75:269–278. 10.1007/10.1177/08465371231190807 37635274 10.1177/08465371231190807

[2] Filippucci E, Di Geso L, Grassi W (2014) Progress in imaging in rheumatology. Nat Rev Rheumatol 10:628–634. 10.1007/10.1038/nrrheum.2014.145 25201383 10.1038/nrrheum.2014.145

[3] Botchu R, Gupta H (2021) Updates of the imaging of musculoskeletal problems. J Clin Orthop Trauma 22:101612. 10.1007/10.1016/j.jcot.2021.101612 34631415 10.1016/j.jcot.2021.101612 PMC8479250

[4] Graña Gil J, Moreno Martínez MJ, Carrasco Cubero MDC (2024) Delphi consensus on the use of telemedicine in rheumatology: RESULTAR study. Reumatol Clin (Engl Ed) 20:254–262. 10.1016/j.reumae.2024.05.005 38821741 10.1016/j.reumae.2024.05.005

[5] Bolster MB, Kolfenbach J, Poeschla A et al (2023) Incorporating telemedicine in rheumatology fellowship training programs: needs assessment, curricular intervention, and evaluation. Arthritis Care Res (Hoboken) 75:2428–2434. 10.1002/acr.25165 37232060 10.1002/acr.25165

[6] Yemeshev Y, Nurmashev B, Zimba O, Kocyigit BF (2025) Clinical implications of teleradiology in rheumatic and musculoskeletal diseases: improving rheumatic care. Rheumatol Int 45:51. 10.1007/s00296-025-05810-w 39945826 10.1007/s00296-025-05810-w PMC11825612

[7] Zimba O, Gasparyan AY (2021) Social media platforms: a primer for researchers. Reumatologia 59:68–72. 10.1007/10.5114/reum.2021.102707 33976459 10.5114/reum.2021.102707 PMC8103414

[8] Myeoung BJ, Park JH, Lee BJ et al (2024) Social media has become a mainstream source of medical information for patients with rheumatic diseases: a cross-sectional survey of patients. Rheumatol Int 44:2159–2166. 10.1007/10.1007/s00296-024-05634-0 38850324 10.1007/s00296-024-05634-0

[9] Mukhamediyarov M, Nurmashev B, Bekaryssova D (2024) Analysis of the quality and credibility of health-related content on YouTube: cross-sectional study. Cent Asian J Med Hypotheses Ethics 5:269–278. 10.47316/cajmhe.2024.5.4.03

[10] Zengin O, Onder ME (2021) Educational quality of YouTube videos on musculoskeletal ultrasound. Clin Rheumatol 40:4243–4251. 10.1007/10.1007/s10067-021-05793-6 34059985 10.1007/s10067-021-05793-6 PMC8166370

[11] Yoon J, Cho SK, Choi SR et al (2025) Expert consensus on developing information and communication Technology-Based patient education guidelines for rheumatic diseases in the Korea. J Korean Med Sci 40:e67. 10.1007/10.3346/jkms.2025.40.e67 39763311 10.3346/jkms.2025.40.e67 PMC11707660

[12] Kocyigit BF, Akaltun MS (2019) Does YouTube provide high quality information? Assessment of Secukinumab videos. Rheumatol Int 39:1263–1268. 10.1007/10.1007/s00296-019-04322-8 31069444 10.1007/s00296-019-04322-8

[13] Zimba O, Gasparyan AY, Qumar AB (2024) Ethics for disseminating Health-Related information on YouTube. J Korean Med Sci 39:e93. 10.1007/10.3346/jkms.2024.39.e93 38412615 10.3346/jkms.2024.39.e93 PMC10896703

[14] Sui W, Sui A, Rhodes RE (2022) What to watch: practical considerations and strategies for using YouTube for research. Digit Health 8:20552076221123707 36105625 10.1177/20552076221123707 PMC9465614

[15] Zhaksylyk A, Yessirkepov M, Akyol A, Kocyigit BF (2024) YouTube is a source of information on public health ethics. J Korean Med Sci 39:e61. 10.3346/jkms.2024.39.e61 38412608 10.3346/jkms.2024.39.e61 PMC10896704

[16] Etzel CM, Bokshan SL, Forster TA, Owens BD (2022) A quality assessment of YouTube content on shoulder instability. Phys Sportsmed 50:289–294. 10.1080/00913847.2021.1942286 34121601 10.1080/00913847.2021.1942286

[17] Adorisio O, Silveri M, Torino G (2021) Evaluation of educational value of YouTube videos addressing robotic pyeloplasty in children. J Pediatr Urol 17. 10.1016/j.jpurol.2020.12.025.:390.e1-390.e4 10.1016/j.jpurol.2020.12.025 33558173

[18] Azak M, Şahin K, Korkmaz N, Yıldız S (2022) YouTube as a source of information about COVID-19 for children: content quality, reliability, and audience participation analysis. J Pediatr Nurs 62:e32–e38. 10.1016/j.pedn.2021.06.024 34247879 10.1016/j.pedn.2021.06.024 PMC8812823

[19] Onder ME, Zengin O (2021) YouTube as a source of information on gout: a quality analysis. Rheumatol Int 41:1321–1328. 10.1007/s00296-021-04813-7 33646342 10.1007/s00296-021-04813-7 PMC7917371

[20] Kocyigit BF, Akyol A (2021) YouTube as a source of information on COVID-19 vaccination in rheumatic diseases. Rheumatol Int 41:2109–2115. 10.1007/s00296-021-05010-2 34562126 10.1007/s00296-021-05010-2 PMC8475344

[21] Korkmaz M, Altin YF, Yagci TF, Korkmaz MD, Akgul T (2024) Is YouTube a reliable and quality source on unilateral biportal endoscopic spine surgery?? A Cross-Sectional study. World Neurosurg 187:e181–e188. 10.1016/j.wneu.2024.04.063 38642831 10.1016/j.wneu.2024.04.063

[22] Eksi Ozsoy H (2021) Evaluation of YouTube videos about smile design using the DISCERN tool and journal of the American medical association benchmarks. J Prosthet Dent 125:151–154. 10.1016/j.prosdent.2019.12.016 32085870 10.1016/j.prosdent.2019.12.016

[23] Karataş L, Utkan Karasu A, Demirsoy N (2024) Is YouTube a sufficient and reliable source to inform patients about cardiac rehabilitation?? A Cross-sectional study. J Cardiopulm Rehabil Prev 44:239–247. 10.1097/HCR.0000000000000864 38875164 10.1097/HCR.0000000000000864

[24] Sankowski AJ, Lebkowska UM, Cwikła J, Walecka I, Walecki J (2013) The comparison of efficacy of different imaging techniques (conventional radiography, ultrasonography, magnetic resonance) in assessment of wrist joints and metacarpophalangeal joints in patients with psoriatic arthritis. Pol J Radiol 78:18–29. 10.12659/PJR.883764 23494635 10.12659/PJR.883764 PMC3596142

[25] Longo UG, de Sire A, De Salvatore S et al (2024) Imaging of glenohumeral osteoarthritis: reliability and reproducibility of radiological classifications. J Back Musculoskelet Rehabil 37:1729–1739. 10.3233/BMR-240187 39093064 10.3233/BMR-240187 PMC11613007

[26] Weaver JS, Omar I, Mar W et al (2022) Magnetic resonance imaging of rheumatological diseases. Pol J Radiol 87:e93–e112. 10.5114/pjr.2022.113390 35280946 10.5114/pjr.2022.113390 PMC8906181

[27] Ożga J, Mężyk E, Kmiecik W, Wojciechowski W, Żuber Z (2024) Magnetic resonance imaging of the musculoskeletal system in the diagnosis of rheumatic diseases in the pediatric population. Reumatologia 62:196–206. 10.5114/reum/190262 39055724 10.5114/reum/190262 PMC11267661

[28] Krishnan KS, Raju G, Shawkataly O (2021) Prevalence of Work-Related musculoskeletal disorders: psychological and physical risk factors. Int J Environ Res Public Health 18:9361. 10.3390/ijerph18179361 34501950 10.3390/ijerph18179361 PMC8430476

[29] Jarab AS, Al-Qerem W, Abu Heshmeh SR et al (2024) Determinants of poor health-related quality of life among outpatients with rheumatoid arthritis in Jordan. PLoS ONE 19:e0312557. 10.1371/journal.pone.0312557 39441805 10.1371/journal.pone.0312557 PMC11498696

[30] Kaplan K, Solak Y (2023) Evaluation of YouTube videos on hepatocellular carcinoma. J Korean Med Sci 38:e50. 10.3346/jkms.2023.38.e50 36808545 10.3346/jkms.2023.38.e50 PMC9941019

[31] Kocyigit BF, Nacitarhan V, Koca TT, Berk E (2019) YouTube as a source of patient information for ankylosing spondylitis exercises. Clin Rheumatol 38:1747–1751. 10.1007/s10067-018-04413-0 30645752 10.1007/s10067-018-04413-0

[32] Sasse M, Ohrndorf S, Palmowski A et al (2023) Digital health information on autoinflammatory diseases: a YouTube quality analysis. Rheumatol Int 43:163–171. 10.1007/s00296-022-05243-9 36374326 10.1007/s00296-022-05243-9 PMC9839787

[33] Richardson MA, Park W, Bernstein DN, Mesfin A (2022) Analysis of the quality, reliability, and educational content of YouTube videos concerning spine tumors. Int J Spine Surg 16:278–282. 10.14444/8215 35444036 10.14444/8215 PMC9930652

[34] Zimba O, Gasparyan AY (2023) Designing, conducting, and reporting survey studies: A primer for researchers. J Korean Med Sci 38:e403. 10.3346/jkms.2023.38.e403 38084027 10.3346/jkms.2023.38.e403 PMC10713437

